# Stress at Home and Female Gender Are Significantly Associated With Non-adherence and Poor Illness Perception Among Patients With Rheumatoid Arthritis

**DOI:** 10.7759/cureus.25835

**Published:** 2022-06-10

**Authors:** Farzana Hashmi, Muhammad Haroon, Saadat Ullah, Sadia Asif, Saba Javed, Zaid Tayyab

**Affiliations:** 1 Department of Rheumatology, Fatima Memorial Hospital (FMH) College of Medicine and Dentistry, Lahore, PAK

**Keywords:** drug compliance, illness perception, stress, gender, nonadherence, adherence, rheumatoid arthritis

## Abstract

Aims

We aimed to assess the level of non-adherence and poor illness perception among rheumatoid arthritis (RA) patients. Additionally, we examined their associations with clinical indicators and outcomes.

Methods

This was a cross-sectional study conducted using data collected at the time of patient enrolment in the Pakistan Registry of Rheumatic Diseases (PRIME) registry. A wide range of clinical variables was studied. To measure adherence, we used the Urdu version of the General Medication Adherence Scale (GMAS), which has recently been validated in RA patients. A Brief Illness Perception Questionnaire (BIPQ) was used to measure illness perception.

Results

The data of consecutive 320 RA patients were reviewed. Thirty-six percent of the cohort (n=116) was noted to have non-adherence. On multiple logistic regression analysis, a significant association of non-adherence was noted with moderate-to-severe stress (odds ratio {OR}: 1.85, confidence interval {CI}: 1.04-3.2), DAS-28 scores (OR: 1.83, CI: 1.52-2.21), Health Assessment Questionnaire (HAQ) scores (OR: 1.77, CI: 1.07-2.92), and deformed joint counts (OR: 1.30, CI: 1.15-1.46). Additionally, non-adherence due to "patient behavior" had a significant association with the male gender (OR: 0.48, CI: 0.26-0.87), unemployment (OR: 1.82, CI: 1.07-3.10), and stress at home (OR: 2.17, CI: 1.35-3.49). Twenty-six percent of the cohort (n=86) was noted to have the most negative illness perception, and on multiple logistic regression analysis, it was significantly associated with male gender (OR: 0.24, CI: 0.11-0.53), age of onset of arthritis (OR: 0.96, CI: 0.94-0.99), and worse HAQ scores (OR: 3.7, CI: 2.2-6.1).

Conclusions

Important adverse factors contributing to non-adherence and negative illness perception highlighted in this study were stress at home, female gender, and younger age of patients.

## Introduction

Rheumatoid arthritis (RA) is a chronic inflammatory joint illness that causes persistent joint discomfort and dysfunction, resulting in a considerable reduction in a person's quality of life. Despite substantial evidence of therapeutic efficacy, adherence to treatment in RA patients remains low, ranging from 30% to 80% [1]. Therefore, a better understanding of medication adherence remains an important evolving factor in achieving better long-term disease outcomes. Adherence by definition is “the degree to which a person’s behavior compliant with agreed recommendations from a health care provider” [2,3]. On the other hand, non-adherence can be defined as any deviation from the recommended doses or duration of a prescribed treatment regimen. One of the most distressing drug-related complications from a patient’s end is drug resistance, as this reduces drug performance, slows down recovery from symptoms, allows disease progression, and affects the quality of life [4].

It is important to evaluate and measure drug adherence to disease-modifying drugs (DMARDs), as improving adherence to these drugs may improve the effectiveness of drug treatment in rheumatic diseases. There is a lack of data about DMARDs adherence among Pakistani patients. Poor medication adherence has many causes, that include socioeconomic elements, treatment-related factors, patient and disease-related factors, and problems related to the health care team [5]. Treatment adherence and the outcome of the disease can be improved by identifying non-adherence factors and incorporating interventions to address specific barriers. Several methods have been proposed and used to assess treatment adherence, for example, direct or indirect methods [5]. Specific methods include direct treatment, and monitoring drug levels and/or metabolites in blood or urine samples. It can be easily done but it delays important boundaries of being aggressive and costly. Indirect methods include the use of prescriptive prescriptions, pill dosages, and patient medication logs, but the most commonly used method of reporting is using a questionnaire. Adherence to the medication of patients with RA is traditionally considered low [1]. Limited information is available on the indicators and the outcomes of patients with non-adherence to medications in Pakistani RA patients.

Illness perceptions are the beliefs that patients have developed about their illness, and these are important predictions of emotional and behavioral reactions to many illnesses. Studies examining the predictive value of illness perception to treatment adherence and illness management behaviors have shown mixed results [6]. Overall, however, the literature on the impact of disease perception suggests that these are important in preventing and controlling illness [7]. The concept of illness perception shows how patients view their disease in terms of cause, previous experience, symptoms, expectations of the recovery process, and coping behaviors [8].^ ^In the context of RA, the patient's perception of patients with arthritis can affect their adaptability to disease, treatment efficacy, and arthritis-related problems with pain and discomfort [9,10]. Patients with chronic diseases and their poor understanding are associated with poor behavior, dysfunction, and poor outcomes [11].

The goal of our study is to evaluate the extent of non-adherence and its relationship to clinical indicators and outcomes using validated measures in a large consecutive Pakistani RA population. In addition, we measured the illness perception using additional proven tools to help us better understand the concept.

## Materials and methods

A cross-sectional study was conducted using data collected during patient enrollment in the Pakistan Registry of Rheumatic Diseases (PRIME) registry. It is an independent, large, anticipated group study, started in 2019 at Fatima Memorial Hospital (FMH) Lahore; it includes patients diagnosed with RA, ankylosing spondylitis, psoriatic arthritis, and systemic lupus erythmatosus by a rheumatologist, and is actively followed. Written informed consent from patients and ethical approval of study obtained from the Institutional Review Board of FMH College of Medicine and Dentistry (approval no. FMH-08-2020-IRB-784-M). We examined the data of patients with RA. The clinical variables assessed were age, sex, tobacco habits, obesity, educational status, marital status, associated comorbidities, and duration of disease (using the Charlson comorbidity index). Participants' educational status was determined by whether or not they had completed secondary school (high school). Activity and severity of disease were assessed according to internationally agreed definitions, such as swollen joints count, tender joints count, and disease activity score-28 (DAS-28). All participants were directly interviewed during the patient enrollment session about the presence of emotional and psychological stress at home and rated it from one to three (mild, moderate, severe).

To measure adherence, the General Medication Adherence Scale (GMAS) tool with the Urdu version was used in the study [12]. It consists of a total of 11 questions along with four possible answers, and the list of questions is divided into three segments. The first segment measured adherence based on patient behavior, the second segment measured adherence in line with comorbidity and pill load, and the third segment measured adherence based on affordability and cost. Each item has an individual score of zero to three. A total of 33 points for all three sections provided adherence for a patient and was further subdivided into adherent, i.e, having a score ≥27 points, and non-adherent, i.e., with ≤26 points. GMAS on 27 points distinguished between adherence and non-adherence. This questionnaire has recently been validated in Pakistan for RA patients [13].

The Brief Illness Perception Questionnaire (BIPQ) is a short and simple version of the Illness Perception Questionnaire (IPQ). It comprises of total nine-item scale and is designed to quickly assess mental and emotional illnesses [14]. Each item scores on an ordinal scale from zero to 10 which produces a total score of zero to 80, and high scores indicate a higher negative illness representation which means the patient considers the disease to be very dangerous. It has the advantage of being short and easy to understand. It has been used in a variety of adult, and pediatric groups and adolescents [15,16]. To facilitate the interpretation of outcomes in daily clinical practice and to identify patients with a negative perception of illness, we have divided BIPQ scores using the 75th interquartile range (IQR) scores as the cutoff, as previously described [17].

Using the SPSS software version 25.0 (Armonk, NY: IBM Corp.) statistical analysis was performed. Significance was considered as p <0.05 (two-tailed). Chi-square arithmetic (χ^2^) was used to examine the distribution of class variants, and Student t-tests were used to analyze additional variables. We applied the odds ratio (OR) and the associated confidence interval (CI) to measure the correlation between the various variables. The association of clinical variables with the various cutoffs of GMAS and BIPQ scores was determined using univariate and multivariate regression. Factors for statistically significant analysis at the 0.25 level were entered into the multivariable model. The model was then reduced to a backward regression until the remaining results were significant at the 0.05 level. The regression coefficient was obtained from this final model.

## Results

The sample frame consisted of consecutive 320 RA patients enrolled in the PRIME registry (mean age 37.4±13.4 years, 74% female, disease duration of 73±68 months, 30% rural residents, 32.5% had low education status of ≤primary school, and 35% of the cohort was employed) was reviewed. Thirty-six percent of the cohort (n=116) was noted to have non-adherence. Gender, age, age of onset of RA, disease duration, marital status, and rural living was not statistically associated with adherence status; however, the borderline association of unemployment (p=0.18) and low education status (p=0.08) was noted with non-adherence status (Table 1).

**Table 1:** Descriptive characteristics of non-adherent RA patients, and their comparison with adherent RA patients. Data are presented as percentage (number) and median±standard deviation. DAS-28: disease activity score-28; HAQ: Health Assessment Questionnaire; BMI: body mass index

Variables	Patients with good adherence	Patients with poor adherence	p-Value
Gender (male)	27 (56)	24 (28)	0.59
Married	60.8 (124)	60.3 (70)	1.0
Rural	29 (59)	32 (37)	0.61
Low education status	29 (59)	39 (45)	0.08
Unemployed	62 (127)	70 (81)	0.18
Stress (moderate-to-severe)	41 (83)	63 (73)	<0.001
Age (years)	37.8±13.8	36.8±12.8	0.52
Disease duration (months)	75±77	69±51	0.45
Age of onset (years)	31±12	30±12	0.66
DAS-28	2.7±1.4	4.5±1.6	<0.001
HAQ	0.76±0.53	1.11±0.60	<0.001
BMI	29±5.3	29±5.6	0.74
Deformed joint counts	1.2±1.8	3.4±3.3	<0.001
Comorbidity index	1.7±1.3	2.2±1.6	0.005

There were statistically significant associations of moderate-to-severe stress at home (p≤0.001), worse Health Assessment Questionnaire (HAQ) (p<0.001), deformed joint counts (p<0.001), higher comorbidity index (p<0.005), and poor disease control (DAS-28 status, p<0.001) with non-adherence. On multiple logistic regression analysis, a significant association of non-adherence was noted with moderate-to-severe stress (OR: 1.85, CI: 1.04-3.2, p=0.03), DAS-28 scores (odds ratio {OR}: 1.83, confidence interval {CI}: 1.52-2.21, p<0.001), HAQ scores (OR: 1.77, CI: 1.07-2.92, p=0.02), and deformed joint counts (OR: 1.30, CI: 1.15-1.46, p<0.001) (Table 2).

**Table 2:** Univariate and multivariate (adjusted simultaneously for variables shown) associations of different clinical variables with non-adherence. OR: odds ratio; CI: confidence interval; DAS: disease activity score; HAQ: Health Assessment Questionnaire

Variables	Univariate model			Multivariate model		
	OR	95% CI	p-Value	OR	95% CI	p-Value
Low education status	1.55	0.96-2.5	0.07	-	-	-
Unemployed	1.4	0.86-2.2	0.17	-	-	-
Stress (moderate-to-severe)	2.47	1.54-3.95	<0.001	1.85	1.04-3.2	0.03
DAS-28	2.04	1.70-2.44	<0.001	1.83	1.52-2.21	<0.001
HAQ	2.92	1.91-4.47	<0.001	1.77	1.07-2.92	0.02
Deformed joint counts	1.40	1.26-1.56	<0.001	1.30	1.15-1.46	<0.001
Comorbidity index	1.24	1.05-1.45	0.008	-	-	-

We further examined the concept of non-adherence among our cohort across three domains or sections of the GMAS questionnaire individually, namely non-adherence due to patient behavior, comorbidity and pill burden, and cost. Firstly, by using univariate and subsequent multivariate regression analysis, we noted that non-adherence related to patient behavior had a significant negative association with the male gender (OR: 0.48, CI: 0.26-0.87, p=0.01), positive association with unemployment (OR: 1.82, CI: 1.07-3.10, p=0.02), stress at home (OR: 2.17, CI: 1.35-3.49, p=0.001), poor disease control indicated by worse DAS-28 (OR: 1.15, CI: 1.00-1.33, p=0.050), and worse HAQ scores (OR: 1.83, CI: 1.19-2.81, p=0.005) (Table 3).

**Table 3:** Univariate and multivariate associations of different clinical variables with behavior-related non-adherence. The following variables were included in the final regression analysis: gender, marital status, employed, stress, comorbidity index, deformed joint counts, HAQ, and DAS-28. OR: odds ratio; CI: confidence interval; DAS: disease activity score; HAQ: Health Assessment Questionnaire

Variables	Univariate model			Multivariate model		
	OR	95% CI	p-Value	OR	95% CI	p-Value
Gender (male)	0.59	0.35-0.99	0.04	0.48	0.26-0.87	0.01
Age	0.99	0.98-1.01	0.62	-	-	-
Marital status (married)	1.33	0.88-2.19	0.15	-	-	-
Rural residence	1.06	0.65-1.73	0.79	-	-	-
Low education status	0.90	0.55-1.46	0.90	-	-	-
Employed	1.32	0.83-2.09	0.23	1.82	1.07-3.10	0.02
Disease duration	0.99	0.99-1.00	0.63	-	-	-
Age of onset	0.99	0.97-1.01	0.75	-	-	-
Stress (moderate-to-severe)	2.37	1.51-3.72	<0.001	2.17	1.35-3.49	0.001
DAS-28	1.25	1.10-1.43	0.001	1.15	1.00-1.33	0.050
HAQ	2.03	1.37-3.03	<0.001	1.83	1.19-2.81	0.005
Deformed joint counts	1.16	1.06-1.26	<0.001	-	-	-
Comorbidity index	1.09	0.94-1.27	0.24	-	-	-

Secondly, univariate and subsequent multivariate regression analysis showed that non-adherence due to comorbidity and pill burden was associated with the age of onset of rheumatoid arthritis (OR: 1.02, CI: 1.00-1.04, p=0.006), worse DAS-28 (OR: 1.18, CI 1.01-1.37, p=0.03), and worse HAQ (OR: 1.81, CI: 1.17-2.81, p=0.008) (table in Appendix 1). Thirdly, univariate and subsequent multivariate regression analysis showed that cost-related non-adherence had no significant association with patient-related demographics and traits, but was noted to have a significant association with worse DAS-28 (OR: 1.26, CI: 1.08-1.47, p=0.002) and worse HAQ scores (OR: 2.17, CI: 1.39-3.40, p=0.001) (table in Appendix 2). The mean total BIPQ score of the cohort was 62±8.8. As described above, BIPQ scores were dichotomized to identify patients with the most negative illness perception using the 75th IQR as the cutoff, and in our study cohort, this cutoff was 68. Twenty-six percent of the cohort (n=86) was noted to have the most negative illness perception (BIPQ score of >68), and Table 4 provides descriptive characteristics of RA patients with and without the most negative illness perception. There was a significantly negative association between male gender, age, and age of onset of arthritis, along with a significantly positive association between worse disease activity (DAS-28) and poor HAQ scores with the most negative illness perception (p<0.05) (Table 4).

**Table 4:** Univariate and multivariate associations of different clinical variables with comorbidity-related non-adherence. The following variables were included in the final regression analysis: age, marital status, low education status, employed, age of onset of arthritis, stress, comorbidity index, deformed joint counts, HAQ, and DAS-28. OR: odds ratio; CI: confidence interval; DAS: disease activity score; HAQ: Health Assessment Questionnaire

Variables	Univariate model			Multivariate model		
	OR	95% CI	p-Value	OR	95% CI	p-Value
Gender (male)	0.95	0.56-1.59	0.85	-	-	-
Age	1.01	0.99-1.03	0.10	-	-	-
Marital status (married)	1.79	1.10-2.89	0.01	-	-	-
Rural residence	1.32	0.80-2.16	0.26	-	-	-
Low education status	1.43	0.88-1.32	0.13	-	-	-
Employed	0.74	0.45-1.20	0.22	-	-	-
Disease duration	0.99	0.99-1.00	0.35	-	-	-
Age of onset	1.02	1.00-1.04	0.01	1.02	1.00-1.04	0.006
Stress (moderate-to-severe)	1.53	0.97-2.43	0.06	-	-	-
DAS-28	1.24	1.09-1.43	0.001	1.18	1.01-1.37	0.03
HAQ	2.06	1.38-3.09	<0.001	1.81	1.17-2.81	0.008
Deformed joint counts	1.14	1.05-1.24	0.002	-	-	-
Comorbidity index	1.05	0.90-1.23	0.47	-	-	-

On multiple logistic regression analysis, a significant protective association between male gender (OR: 0.24, CI: 0.11-0.53, p<0.001) and age of onset of arthritis (OR: 0.96, CI: 0.94-0.99, p=0.01) along with the significant association of worse HAQ scores (OR: 3.7, CI: 2.2-6.1, p<0.001) was noted with the most negative illness perception (Table 5).

**Table 5:** Univariate and multivariate (adjusted simultaneously for variables shown) associations of different clinical variables of RA patients with and without most negative illness perception (as per BIPQ). OR: odds ratio; CI: confidence interval; BIPQ: Brief Illness Perception Questionnaire; RA: rheumatoid arthritis; DAS: disease activity score; HAQ: Health Assessment Questionnaire

Variables	Univariate model			Multivariate model		
	OR	95% CI	p-Value	OR	95% CI	p-Value
Gender (male)	0.32	0.16-0.64	0.001	0.24	0.11-0.53	<0.001
Age (years)	0.97	0.95-0.99	0.007	-	-	-
Disease duration	0.99	0.99-1.00	0.29	-	-	-
Age of onset	0.96	0.94-0.99	0.005	0.96	0.94-0.99	0.01
BMI	1.04	0.99-1.09	0.058	-	-	-
Stress (moderate-to-severe)	1.47	0.89-2.42	0.12	-	-	-
DAS-28	1.24	1.07-1.43	0.003	-	-	-
HAQ	3.4	2.1-5.4	<0.001	3.7	2.2-6.1	<0.001
Comorbidity index	1.12	0.96-1.3	0.14	-	-	-

Additionally, we also studied the associations using BIPQ scores as a continuous variable. On multiple logistic regression analyses, similar results were noted. For example, a significant negative association between the male gender, but a positive association between DAS-28 and HAQ was noted with poor illness perception scores (data not shown).

## Discussion

There has been an overall paucity of research regarding the indicators and the outcomes of the patient having non-adherence to medications among Asians, especially Pakistani RA patients. This study has revealed some important demographic characteristics contributing to poor adherence to medications, along with its adverse outcomes. We believe that local beliefs and practices and races have important implications.

From a clinical point of view, the outcomes of this study are important in many ways. Firstly, non-adherence was noted to be common in Pakistani RA patients, as more than one-third of patients were not adherent. There are various reports of drug adherence among RA patients with significant variability ranging from 9% to 80%; however, depending on the geographic area, the definition of adherence applied and the detection tools used to study adherence [18-21]. Reporting yourself is a typical technique to monitor drug adherence since it is easy to carry, takes less time, is flexible, and is low-cost. Medication pill counting, electrical monitoring, rates of refilling prescriptions, and sample testing of drug levels or drug byproducts are some of the other approaches used to assess adherence [22,23].

In a recent Korean study, only 9.6% of patients with RA were found to be non-adherent to the medications by defining non-adherence if patients skipped ≥6 days of RA medications. However, the authors agree that this particular level of adherence was due to the studied patient's characteristics as they had a good relationship with physicians [24]. Our study, in contrast, was conducted on a standard treatment regimen and included all patients who visit our rheumatology clinics. In Pakistan, methotrexate treatment non-adherence has been reported in 23% of RA patients, and the authors describe neutrality as a violation of any three or more prescribed methotrexate (MTX) doses in the previous eight weeks [25]. Another local study revealed 51% non-adherence in RA patients using the GMAS questionnaire [13].

Secondly, our study reveals gender-related significant disparities; for example, behavior-related non-adherence was noted to be significantly worse among female RA patients on univariate and multivariate analyses after controlling for associated confounders. Many factors that may contribute to treatment non-adherence have been explored in the literature, although the contribution of patient gender on treatment adherence has not been extensively investigated. Some studies investigating chronic treatment adherence have found that women are more adherent than men, while others have reported opposite effects [26,27]. In Pakistan, a previous study did not report significant gender differences concerning methotrexate treatment adherence in RA patients [26]. A study from our neighboring country has evaluated the adherence to therapy in females with RA, and the authors reported a high non-adherence rate among 52% of patients and a high level of dissatisfaction with anti-rheumatic treatment among 68% of women [28]. In addition, our study revealed significant differences in overall BIPQ scores between female and male patients (female 63±8 vs. male 59±10, p=0.002), meaning that women perceived RA as a highly threatening disease. The implementation of BIPQ in our study shows that patients' perceptions of RA are in concordance with patients’ gender, while no correlation was found in the patient's marital status, rural/urban settlements, educational status, employment status, illness, and disease duration. Evaluation of illness perception is warranted in daily clinical practice, as the way patients relate to the whole course of the disease has a direct impact on their behavior, compliance to treatment, and outcome of the disease. Therefore, an individual approach is needed to improve adherence to RA medications.

Thirdly, stress at home was identified as a major risk factor for non-compliance. Although there are many factors are to contribute to non-adherence in chronic diseases such as RA but little is known about the impact of domestic stress on non-adherence among Asian patients of Pakistani origin. This may contribute to the development of prevention strategies and interventions to manage stress levels in vulnerable populations. The plausible explanations that how perceived stress at home could influence compliance are: stress with a high level is associated with low cognitive function, and stress can impair learning and memory [29,30]. The limitation of this study is the lack of information about long-term stress levels in our patients. If such data were collected, it may contribute to a better understanding of the relationship between RA and stress. Fourth, our study showed that one of the most important factors associated with poor adherence and poor perception of illness was the age of the patient, and older patients were more willing to comply compared to younger ones. Young people often participate in work and social activities, and as a result, find themselves busy with work and family responsibilities which is a possible explanation.

We realize that our study has some barriers. As an example, as this was not a population-based study, there is a possibility of selection bias, the homogeneity of the group (population of Pakistan) also can be considered as the omission bias, which, along with the predominance of high disease activity within the cohort probably limits the generalizability of the study’s findings; the prevalence of adherence and the perception of illness were examined in one cross-sectional assessment, which is not a perfect study style to investigate its predictors; but, this still provides helpful data that ought to be considered in alternative future studies. Likewise, we didn’t probe other threat factors similar as sleep disturbance, low income, menopause, depression, anxiety, and physical inactivity, which can confound these effects. The strengths of our research include the following: we have included a wide range of demographic data, clinical features, patient outcome measures, and a variety of disease activity indicators, which has allowed us to prob the factors that may be contributing. To minimize selection bias, we tried to recruit all consecutive cases and standardize the research process, data from only one institution were included when all patients were reviewed by a single team of qualified rheumatologists.

## Conclusions

We conclude that poor compliance and negative illness perception remain a common issue among RA patients, and our study further confirms its adverse clinical outcomes. Important adverse factors highlighted in this study were stress at home, female gender, and younger age of patients. In resource-poor countries where access to biological therapies is already limited, behavior-related interventions are required to target at-risk populations to improve adherence to commonly prescribed anti-rheumatic drugs.

## Appendices

Appendix 1

**Table 6:** Univariate and multivariate associations of different clinical variables with comorbidity-related non-adherence. OR: odds ratio; CI: confidence interval; DAS: disease activity score; HAQ: Health Assessment Questionnaire

Variables	Univariate model			Multivariate model		
	OR	95% CI	p-Value	OR	95% CI	p-Value
Gender (male)	0.95	0.56-1.59	0.85	-	-	-
Age	1.01	0.99-1.03	0.10	-	-	-
Marital status (married)	1.79	1.10-2.89	0.01	-	-	-
Rural residence	1.32	0.80-2.16	0.26	-	-	-
Low education status	1.43	0.88-1.32	0.13	-	-	-
Employed	0.74	0.45-1.20	0.22	-	-	-
Disease duration	0.99	0.99-1.00	0.35	-	-	-
Age of onset	1.02	1.00-1.04	0.01	1.02	1.00-1.04	0.006
Stress (moderate-to-severe)	1.53	0.97-2.43	0.06	-	-	-
DAS-28	1.24	1.09-1.43	0.001	1.18	1.01-1.37	0.03
HAQ	2.06	1.38-3.09	<0.001	1.81	1.17-2.81	0.008
Deformed joint counts	1.14	1.05-1.24	0.002	-	-	-
Comorbidity index	1.05	0.90-1.23	0.47	-	-	-

Appendix 2 

**Table 7:** Univariate and multivariate associations of different clinical variables with cost-related non-adherence. OR: odds ratio; CI: confidence interval; DAS: disease activity score; HAQ: Health Assessment Questionnaire

Variables	Univariate model			Multivariate model		
	OR	95% CI	p-Value	OR	95% CI	p-Value
Gender (male)	0.74	0.42-1.30	0.30	-	-	-
Age	0.99	0.97-1.01	0.52	-	-	-
Marital status (married)	0.76	0.47-1.23	0.27	-	-	-
Rural residence	1.56	0.94-2.59	0.08	-	-	-
Low education status	1.49	0.91-2.46	0.11	-	-	-
Employed	0.65	0.39-1.10	0.11	-	-	-
Disease duration	0.99	0.99-1.00	0.14	-	-	-
Age of onset	0.99	0.97-1.01	0.63	-	-	-
Stress (moderate-to-severe)	1.14	0.70-1.83	0.58	-	-	-
DAS-28	1.34	1.16-1.54	<0.001	1.26	1.08-1.47	0.002
HAQ	2.67	1.74-4.12	<0.001	2.17	1.39-3.40	0.001
Deformed joint counts	1.11	1.02-1.21	0.01	-	-	-
Comorbidity index	1.05	0.90-1.23	0.47	-	-	-
